# Enhancing Biocompatibility and Biophysical Properties of Three-Dimensional Collagen Scaffolds Using Nonthermal Plasma Treatment

**DOI:** 10.1021/acsbiomaterials.5c02062

**Published:** 2026-02-04

**Authors:** Noof Sulaiman, Mohamed Abdulla, Priya Das, Praveen Kumar Manyam, James Blackwell, Matthew McGrath, Roshan Deen, Andy Ma, Fergal J. O’ Brien, Micheal B. Keogh

**Affiliations:** † School of Medicine, 123320 RCSI Medical University of Bahrain, Royal College of Surgeons in Ireland, P.O. Box 15503, Adliya 228, Kingdom of Bahrain; ‡ Tissue Engineering Research Group Bahrain, School of Postgraduate Studies and Research, Royal College of Surgeons in Ireland, P.O. Box 15503, Adliya 228, Kingdom of Bahrain; § Tissue Engineering Research Group, Department of Anatomy and Regenerative Medicine, 8863 Royal College of Surgeons in Ireland, 123 St. Stephen’s Green, Dublin D02 YN77, Ireland; ∥ Advanced Materials and Bioengineering Research Centre, Royal College of Surgeons in Ireland and Trinity College Dublin, Dublin D02 PN40, Ireland

**Keywords:** biomaterials, biocompatible, collagen scaffold, nonthermal plasma treatment, mechanical strength

## Abstract

Collagen-glycosaminoglycan (CG) scaffolds are extensively utilized in tissue engineering for their excellent biocompatibility and low immunogenicity; however, their poor mechanical stiffness typically requires further physical or chemical modifications to enhance their structural integrity for clinical applications. We investigate the effects of nonthermal plasma (NTP) treatment; an emerging technology commonly used in the biomedical field for surface modifications, sterilization, and wound healing. A comprehensive analysis is conducted to evaluate the surface characteristics, biophysical properties, and biocompatibility of the 3D CG scaffolds treated with NTP for 2 and 5 min, compared with untreated controls. Histological and SEM analyses demonstrated thickening of the scaffold pore struts and an increase in porosity, while Energy Dispersive X-ray Spectroscopy (EDS) and Fourier transform infrared spectroscopy (FTIR) confirmed that the native chemical composition of the scaffolds remained intact and unchanged following NTP exposure. Post-treatment, the scaffolds exhibited increased hydrophilicity demonstrated by a reduced contact angle. Mechanical testing showed significant improvements in the scaffold’s compression modulus, with increases of approximately 16.7% and 14.5% for 2 min and 5 min treatments, respectively (*p* < 0.05). *In vitro* biocompatibility assays indicated increased metabolic rates and significantly higher cell numbers in the ADSC-seeded on NTP-treated scaffolds (*p* = 0.001; *p* = 0.02, respectively). Following 21 day osteogenic conditions, both 2 min and 5 min NTP-treated scaffolds exhibited significantly elevated expression of key osteogenic markers, with RUNX2 showing a 9-fold increase at 2 min and an 11-fold increase at 5 min (*p* < 0.001), and Osteocalcin demonstrating increases of 2.5-fold and 2.3-fold, respectively (*p* < 0.01), compared to untreated controls. The enhanced biocompatibility and ability to serve as a supportive matrix that promotes osteogenic lineage commitment observed in the NTP-treated scaffolds suggest that these materials could be effectively utilized as allogenic osteocyte-loaded biomaterials for bone regeneration. Collectively, these results demonstrate that NTP treatment significantly improves the functional performance and mechanical strength of the 3D CG scaffolds, establishing it as an effective approach for enhancing scaffold performance in regenerative medicine applications.

## Graphical abstract

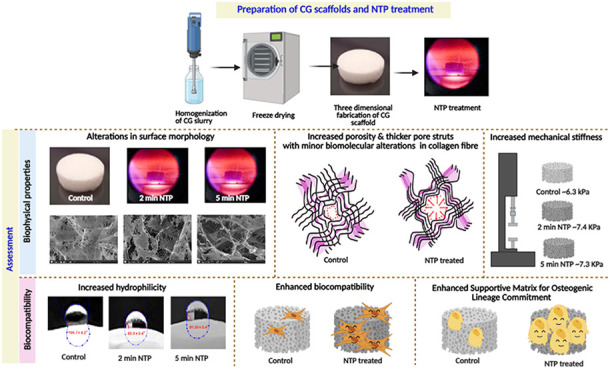

## 1 Introduction

Collagen scaffolds have emerged as a significant biocompatible biomaterial in tissue engineering for advanced wound dressings due to their excellent biocompatibility, bioconductivity, and low immunogenicity.^1,2^ Alone or in combination as a composite with glycosaminoglycans such as chondroitin sulfate or hyaluronic acid, these scaffolds stimulate wound healing by playing the role of a macromolecular ligand or protein mediator, leading to the regulation of tissue homeostasis, cell migration, and cell signaling.^1,3,4^ However, this highly compliant scaffold is vulnerable due to poor mechanical stability and enzymatic degradation.^1,5^ To improve mechanical stability, chemical cross-linkers are employed to effectively cross-link collagen fibers. Commonly used chemical cross-linkers are carbodiimide hydrochloride in combination with *N*-hydroxy-sulfosuccinimide, which effectively enhances the scaffold’s mechanical strength and resistance to enzymatic degradation.^6^ However, they can present potential drawbacks such as cytotoxicity or alterations to the scaffold’s biological properties that require several washing steps. Inadequate washing or residual chemicals can increase toxicity, leading to cell death.^7–9^

Novel technologies without the use of solvents, such as melt molding and fused deposition modeling, have been introduced to improve the overall surface and mechanical properties of the scaffolds.^10^ Another such treatment is nonthermal plasma (NTP), typically used to decontaminate and sterilize materials.^11,12^ NTP is generated by ionizing a gas discharge, where the gas is excited into energetic states through mechanisms such as radio frequency, microwave, or electron emitted from a hot filament discharge. Plasma generation is influenced by the geometry of the discharge chamber and gas medium, where variations in particle density and collision frequency regulate the quality and extent of etching, functionalization, and cross-linking.^13^ The charged particles in the plasma are accelerated at relatively low gas temperature and pressure, resulting in a stable, controllable plasma that does not significantly alter the chemical composition or fundamental properties of the treated biomaterial due to its nonthermal nature.^14^ This characteristic ensures the preservation of the material’s intrinsic chemical structure while enabling surface and biophysical modifications. This makes NTP a suitable candidate for treating biomaterials without thermal damage to the bulk structure or material.^15–18^ Bulk modifications involve altering the entire biomaterial throughout its structure rather than just its surface. In contrast, surface modifications target only the outer layer of the material, which is typical of treatments like NTP that modify surface properties without affecting the bulk composition.^17^ The modified surface characteristics often enhance its reactivity, hydrophilicity, and promote chemical bonding.^16,19^ Moreover, the penetrability of plasma through the porous biomaterials enhances cells’ three-dimensional attachment and expansion without toxic chemicals penetrating the sample.^17^ The versatility of controlled plasma modification is seen in surface functionalization that introduces desired functional groups like amide, imide, nitrile, and hydroxyl on the surface of the biomaterial, which directly influence the degradation rate of the biomaterials, as well as effectively sterilize the surface.^15,20,21^ In addition, the surface functionalization tends to enhance binding efficiency, creating a stable bioactive matrix that mimics the native extracellular matrix (ECM).^22^ Several studies have shown that plasma-treated materials are more suitable as biomedical scaffolds or implants as compared to nontreated counterparts. Nitrogen plasma-treated polylactic acid (PLA) scaffolds exhibited enhanced physiochemical and biochemical properties.^12^ The poly­(vinylidene fluoride) (PVDF)-carbon nanohorn (CNH) composites showed better hemocompatibility and less cytotoxicity when treated with nitrogen plasma.^23^ The modified surface area available for cell attachment directly influences cell signaling and cell adhesion by enhancing porosity, thereby supporting the development of a functional tissue microenvironment.^21,24^ For example, studies have shown that functional amide groups on the 2–5 min Ar-NH3/H2 plasma-treated electrospun poly­(l-lactide) (PLLA) scaffolds are reported to have enhanced cell attachment and growth.^25^ Another study by Roh et al. (2017) described a 3-min oxygen and nitrogen plasma treatment of 3D PCL scaffolds, which showed improved cellular adhesion, proliferation, and differentiation of preosteoblasts.^26^ Air and oxygen plasma treatment of 3D-printed PLA-TiAl4V for 6 min enhanced the surface roughness and wettability, thereby increasing its biocompatibility.^27^ Additionally, the long-term stability of plasma-induced changes and their impact on the scaffold’s biological performance need further investigation.^14,28^

Compared to chemical cross-linking and other surface modification methods, NTP treatment offers the advantage of being a quick, one-step,^24^ solvent-free, eco-friendly process that does not introduce potentially cytotoxic residues. It also allows for more precise control over the depth of modification, typically affecting not only the outermost layers of the material but also the inner morphology of the pores. The effects can be highly dependent on treatment parameters such as gas composition, power, treatment time, and pressure.^24,29^ Other NTP studies done on the high-density polyethylene surface also presented increased surface roughness with distinct etch marks and grooves.^30^ The NTP treatment varies in a dose-dependent manner, and most studies focus on varying exposure time between 60 s and 10–15 min; however, exposures of 2–5 min have demonstrated improved outcomes, including enhanced biomaterial stability.^24–26,31,32^

However, little is known about NTP treatments for collagen-based scaffolds. Therefore, in this study, we aim to investigate the biophysical effects of radio frequency tailored nitrogen plasma treatment on collagen glycosaminoglycan scaffolds and its effects on biocompatibility in cell attachment, growth, and adipose-derived stem cell differentiation.

## 2 Materials and Methods

### 2.1 Preparation of CG Scaffolds

Collagen glycosaminoglycan (CG) scaffolds were prepared using optimized freeze-drying techniques.^33^ Briefly, bovine tendon type 1 collagen (0.5% w/v) and shark cartilage chondroitin-6-sulfate (0.05% w/v; Sigma, UK) were blended in acetic acid (0.05 M). The slurry was degassed and subjected to a freeze-dryer. Both the chamber and shelf were cooled at a constant rate of 1 °C min^–1^ to a final temperature of −10 °C. This temperature was maintained for 60 min, and then sublimated under vacuum at 0 °C for the next 17 h to produce a highly porous and homogeneous scaffold sheet.^33^ 8 and 5 mm scaffolds were punched out using sterile biopsy punches.

### 2.2 Nonthermal Plasma Treatment

Plasma cleaner PDC-002 (Harrick Plasma) was employed to treat scaffolds at two different time points: 2 and 5 min. Samples were placed in the vacuum chamber at a radio frequency level of 13.56 MHz, power of 30 W, a pressure of 100 mTorr, and a nitrogen gas flow rate of 49 mL/min. Samples were exposed for 2 and 5 min, respectively, under an applied voltage of 230 V.^16^

### 2.3 Scanning Electron Microscopy (SEM) and Energy Dispersive Spectroscopy (EDS) Analysis

The surface morphology and microstructure of the samples were analyzed using a field-emission scanning electron microscope (FESEM, Quattro S, Thermo Fisher Scientific, USA). Prior to imaging, the scaffolds were sputter-coated (10 nm gold layer; Inovenso SPT-20) at 15 kV voltage. SEM images were acquired using the Everhart–Thornley secondary electron detector (ETD) at accelerating voltages of 10 and 15 kV, with magnifications ranging from 100× to 500×, as required for optimal resolution.

Qualitative and semiquantitative elemental composition was determined by energy dispersive X-ray spectroscopy (EDS, Ultradry, Thermo Fisher Scientific, USA), integrated with the SEM system. The EDS detector collected characteristic X-rays emitted from the sample upon electron beam irradiation. Elemental mapping and point analyses were performed using the associated software to determine the qualitative and semiquantitative distribution of elements across the sample surface.

SEM images were used for pore analysis, and the porosity was measured using elliptical tool functions of ImageJ (Fiji 64). Approximately 60 pores were measured for each SEM image, and the dimensions were computed.^34^

### 2.4 Histological Evaluation of Scaffolds

Scaffolds were fixed in 4% paraformaldehyde for 20 min and processed using a tissue processor. Five-micrometer-thick sections were cut and thoroughly deparaffinized with two changes of xylene for 5 min each. This was followed by rehydration through a graded ethanol series: 100%, 95%, 70%, and 50% ethanol. The slides were then rinsed in distilled water before being stained with five drops of alcian blue reagent to visualize collagen fibers. After staining, sections were dehydrated through ascending alcohol concentrations (70%, 90%, and 100%) and cleared in xylene prior to mounting. Images were captured in an Olympus BX43 bright field and analyzed using ImageJ software (Fiji 64).

### 2.5 Mechanical Testing

Dry compressive testing was performed on 8 mm scaffold samples grouped as untreated and post-NTP treatment for 2 and 5 min (*n* = 25 for each group). Mechanical testing for dry compression was measured using the UniVert mechanical test system (CellScale, Canada) fitted with a 5 N load cell and 10% strain with a stretch dimension of 300 s. An unconfined compression test with unlubricated platens was used. The compressive modulus was calculated by the slope of a linear fit to the stress–strain curve over 2–5% strain.^33^

### 2.6 FT-IR Analysis

Prior to the Fourier transform infrared (FTIR) spectrum analysis, samples were powdered with Potassium bromide (KBr) (7758-02-3, Sigma-Aldrich, UK), and the pellets were recorded on a Bruker Alpha FT-IR spectrophotometer (Germany) with a spectral resolution of 0.5 cm^–1^ in the range between 4000 and 400 cm^–1^. Thirty scans were collected with baseline correction. The spectra have been processed with Origin61 (v6-1052) software.

### 2.7 Contact Angle and Absorption Rate Measurements

For contact angle analysis, scaffolds from control, 2 min, and 5 min NTP treatment groups were placed on a Petri dish. A 3 μL droplet of deionized water was carefully deposited onto each scaffold surface. Contact angle measurements were performed in quadruplicate (*n* = 4) for each group, and the average values were calculated. The contact angle was determined by fitting a B-spline curve around the droplet outline, and the tangent to this curve at the contact point was used to calculate the angle using ImageJ software (Fiji 64).^15,35^

Absorption rate measurements were obtained from video recordings captured at 30 frames per second with a 4K resolution. Aperture settings, including ISO and shutter speed, were dynamically adjusted by the device’s intelligent exposure control algorithms to optimize image quality during acquisition.^36^

### 2.8 Enzymatic Degradation

Enzymatic degradation of the CG scaffold was analyzed. Control, 2 min, and 5 min NTP (*n* = 4) scaffolds were soaked in Dulbecco’s phosphate-buffered saline (DPBS) for 15 min and weighed prior to enzymatic degradation. The samples were immersed in 1.5 mL of DPBS containing collagenase type-A (1 U/mL) and incubated at 37 °C for 48 h. Collagenase from each sample was removed at 1, 2, 3, 4, 24, and 48 h time points, and the wet weight of the samples was recorded. The weight loss was calculated using the formula in eq 1.^37^

1 $$\%Weight\;Loss=\left[\frac{W_{i}-W_{t}}{W_{i}}\right]\times 100$$

(*W*_*t*_ is the residual wet weight at different time points and *W*_*i*_ is the initial wet weight).

### 2.9 Expansion and Cell Seeding of ADSCs

Normal human adipose-derived stem cells (ADSCs; Lonza, Walkersville, MD, USA; cat. PT-5006) were cultured in Dulbecco’s Modified Eagle’s Medium (DMEM) and Ham’s F-12 Nutrient Mixture (11320-074, Gibco, UK), supplemented with 2% ADSC growth supplement (PT-4503, Gibco, UK), 10% FBS (Gibco, UK), 2% penicillin/streptomycin (Sigma-Aldrich, UK), and 1% amphotericin B (Gibco, UK). Cells were passaged at 70–90% confluency and expanded to passage 3.

ADSCs were seeded onto 5 mm scaffolds at 2.5 × 10^5^ cells per side in the groups untreated control, 2 min post-NTP, and 5 min post-NTP scaffolds (*n* = 3). After 20 min of incubation, 750 μL of DMEM/Ham’s F-12 was added per well in a 24-well plate and incubated under standard culture for 24 h (5% CO_2_ at 37 °C).

### 2.10 Alamar Blue Cell Viability Assay

The cell-seeded untreated control, 2 and 5 min NTP constructs (5 mm) were inserted into a 96-well plate with 150 μL media and 15 μL alamarBlue reagent (Thermo Scientific, Germany) and incubated on a rocker for 5 h in the dark at 37 °C. Postincubation, 100 μL of media was collected and the fluorescence was measured using CLARIOstar (BMG Labtech) software V5.40 R3 (excitation: 570 nm and emission: 600 nm). Absorbance readings were corrected by subtracting the blanks to eliminate background interference.

### 2.11 Cell Count Using DAPI

Cell-seeded scaffolds were fixed in 4% paraformaldehyde for 20 min and processed by using a tissue processor. Five-micrometer-thick sections were deparaffinized thoroughly with two changes for xylene, 5 min each, followed by descending hydration through two changes of ethanol: xylene (1:1), 100% ethanol, 95% ethanol, 70% ethanol, 50% ethanol, and 100% distilled water. DAPI solution (10 μL, 300 nM) was added to each section in the dark and incubated for an hour; unbound dye was rinsed with distilled water. Results were determined as the average of five images and shown as the mean ± SD. Images were captured in an Olympus BX43 fluorescent microscope and analyzed using ImageJ software (Fiji 64).

### 2.12 ADSCs Osteogenic Differentiation in CG Scaffold

2.5 × 10^5^ ADSC cells were seeded each on both sides of the scaffolds in the groups untreated control, 2 min, and 5 min NTP constructs. Cell-seeded constructs were incubated under standard conditions (5% CO_2_, 37 °C) in DMEM/Ham’s F12 and then supplemented with osteogenic inducers: 10 nM dexamethasone (Sigma), 50 μg/mL 2-phospho-l-ascorbic acid (Sigma), and 1% glycerol-2-phosphate (Sigma). Media was replenished every 3 days and constructs were cultured for 21 days.

### 2.13 Alizarin Red S Staining

Bone mineralization of the untreated and NTP-treated cell-seeded scaffolds cultured in osteogenic differentiation media was determined by alizarin red staining. The scaffolds were washed in PBS and fixed with 4.0% formaldehyde for 20 min on the 21st day of culture.

The scaffolds were dehydrated through a graded series of alcohol concentrations and subsequently embedded in paraffin wax. Five μm sections were taken using a microtome, and the sections were stained with 40 mmol/L of alizarin red staining solution (pH = 4.2) for 10 min at room temperature. Excess stains were rinsed with deionized water. The stained sections were then imaged using cellSens Imaging Software (Evident, Olympus Life Science Solutions, Japan) (4×).

### 2.14 RNA Isolation and Real-Time PCR for Assessing Expression of Osteogenic-Related Genes

Total RNA was extracted from cell-seeded scaffolds on day 21 using TRIzol, followed by purification with the RNeasy Mini Kit (Qiagen). Total RNA was quantified using the NanoDrop spectrophotometer (Thermo Scientific Multiskan Sky). All samples were assessed for the A260/A280 ratio, where a value >2 indicates high RNA purity.

cDNA amplification and quantification were carried out by using the Applied Biosystems 7500 Real-Time PCR system. Real-Time PCR expression was determined for the following osteogenic markers: Runt-related transcription factor 2 (Runx2), Osteocalcin (OCN), and Glyceraldehyde 3-phosphate dehydrogenase (GAPDH) (Qiagen) as displayed in Table 1. Means and standard deviations (SD) were calculated. All samples were analyzed in duplicate, and the ΔΔCt method was used to calculate gene expression levels normalized to GAPDH values and expressed relative to nonosteogenic controls.

**Table 1:** Primer Sequence Used in RT-PCR

Gene	Primer Sequence
OCN F	ATGATGGGGACCCCACATCCATAG
OCN R	GTGTCGCTCTGCTGGCCTGG
RUNX2 F	CCCAGTATGAGAGTAGGTGTCC
RUNX2 R	GGGTAAGACTGGTCATAGGACC
GAPDH F	GTCTCCTCTGACTTCAACAGCG
GAPDH R	ACCACCCTGTTGCTGTAGCCAA

### 2.15 Statistics

The data were subjected to statistical analysis using R studio (2023.06.2), and IBM SPSS software (v.32.0). Descriptive statistics were used to compute the mean and standard deviation of continuous variables. The Student’s *t*-test was used to assess the statistical difference between the means of two groups and general linear models of ANOVA were used for multiple comparisons. The *p*-values of <0.05 were considered statistically significant. The data presented are expressed as mean ± standard deviation.

## 3 Results

### 3.1 Evaluation of Surface Morphology, Pore Size, and Histological Characteristics

SEM analysis of dried CG scaffolds revealed alterations in surface morphology for the 2 and 5 min NTP-treated groups compared to the controls with pore size increasing and struts appearing thicker with the increased exposure to plasma (Figure 1A). The histological staining of the scaffolds with alcian blue shows evidence of this, where pore struts appear with darker staining of strands post 2 and 5 min NTP treatment when compared to that of controls maybe an indicator of strand thickness (Figure 1B). Pore size analysis was performed on the SEM images of the dry scaffold and the mean values of the area, perimeter, Feret diameter (the longest distance between two points in the selection), and aspect ratio (ratio of major/minor axis of pores) are shown in Figure 1C. Post-hoc analysis showed that the area, perimeter, and Feret diameter of 5 min NTP scaffold were significantly more as compared to the control and 2 min NTP scaffold (*p* < 0.01). The aspect ratio of the scaffolds indicated elongated pores (ratio >1). The aspect ratio of the 2 min NTP scaffold was significantly more than that of the control (*p* = 0.01) and so was the aspect ratio of the 5 min NTP scaffold as compared to the control (*p* = 0.02).

**Figure 1:**
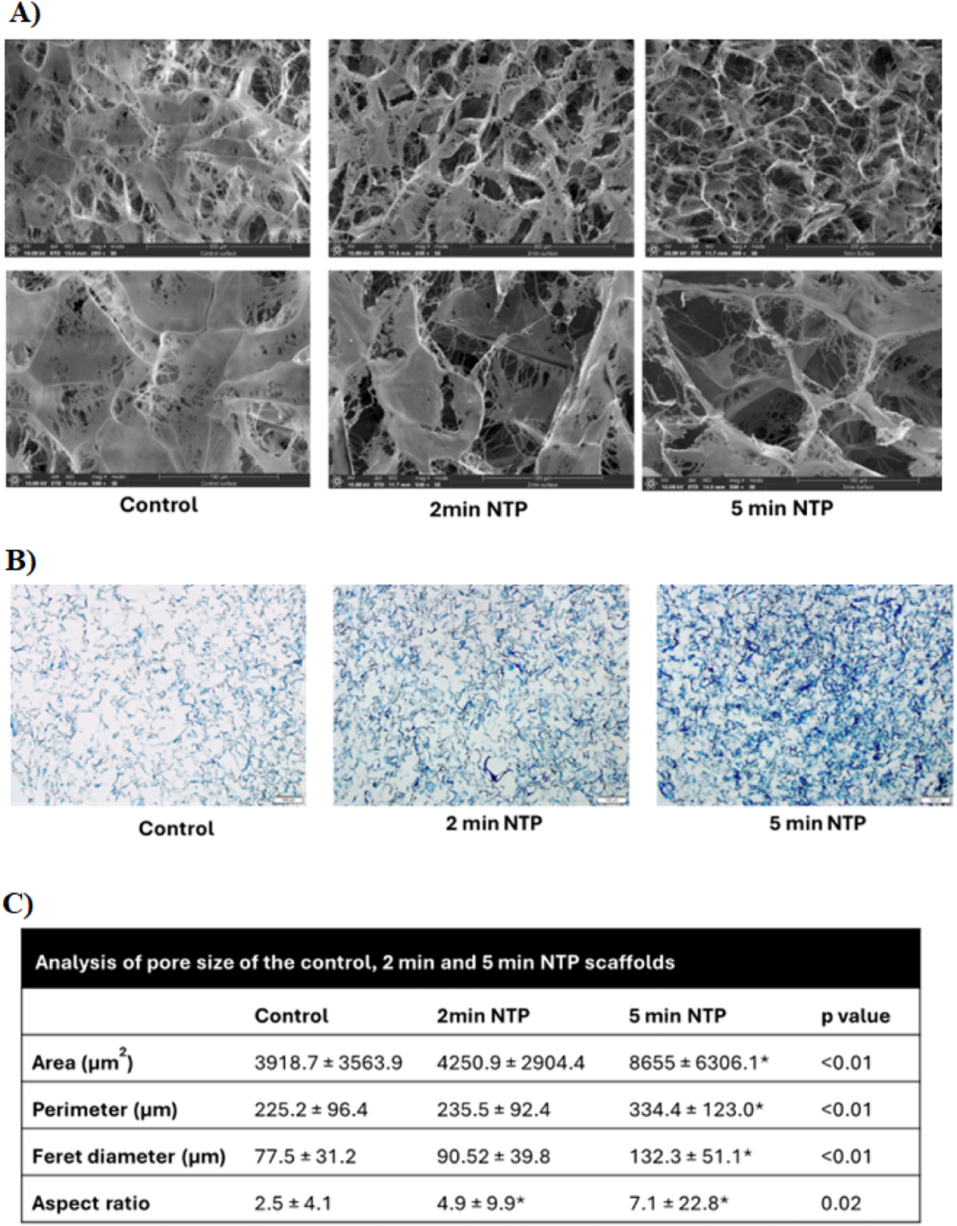
A) Changes in surface and pore morphology as depicted by scanning electron microscope (SEM) images of control, 2 min, and 5 min NTP scaffolds at 200× and 500× magnification; B) Alcian blue staining showing the CG scaffold stained differently for control, 2 min, and 5 min NTP scaffolds. Darker stain can be an indicator of thicker collagen fibers post-NTP. (Scale bar 100 μm); C) Analysis of pore size of the control, 2 min, and 5 min NTP scaffolds, which significantly increase in area seen in the 5 min NTP scaffolds versus control.

### 3.2 NTP-Treated Scaffolds Exhibited Enhanced Compressive Modulus

The compressive modulus of the untreated control scaffold was 6354.2 ± 1131.1 Pa, the 2 min NTP scaffold was 7401.3 ± 1325.2 Pa, and the 5 min NTP scaffold was 7279.0 ± 1948.5 Pa. The mechanical strength significantly improved post-NTP treatment at 2 min (*p* = 0.03) and 5 min (*p* = 0.04), respectively (Figure 2).

**Figure 2:**
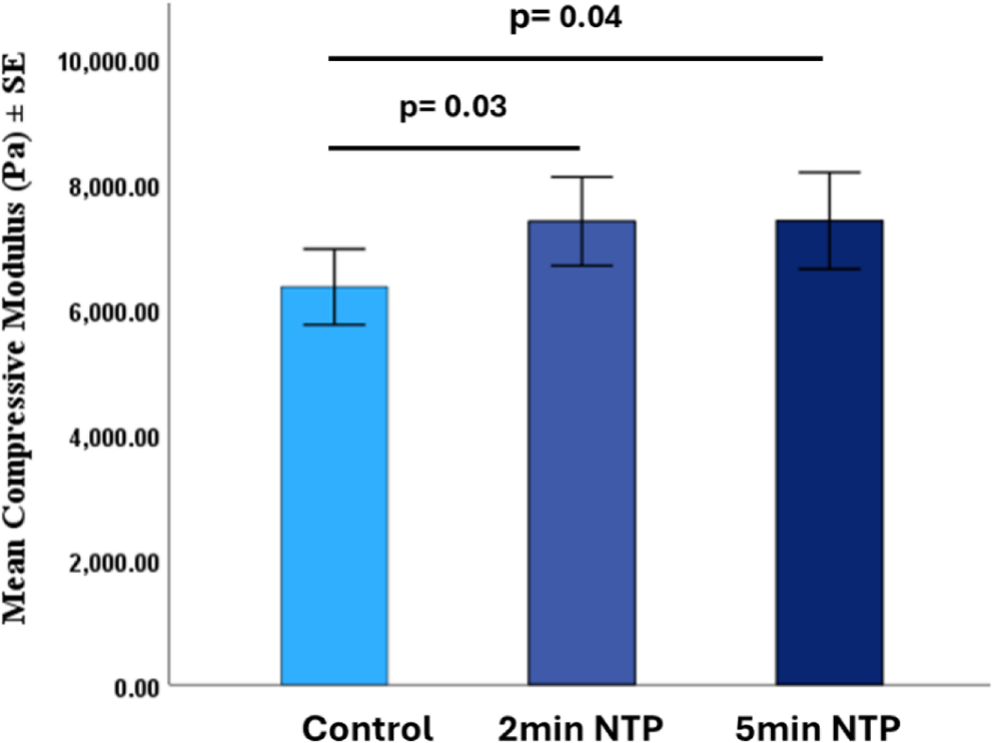
Compressive modulus of dry scaffolds indicating stiffness of the scaffold, with a significant increase in 2 min NTP (7401.3 ± 1325.2 Pa, *p* = 0.03) and 5 min NTP (7279.0 ± 1948.5 Pa, *p* = 0.04) versus control (6354.2 ± 1131.1 Pa).

### 3.3 No Alterations in Chemical Composition and Elemental Profile of Scaffolds Following NTP Treatment

In the FT-IR spectra display, four main characteristic spectral intervals were observed: absorption of amide I v­(C=O) at 1630 cm^–1^, absorption of δ­(CH2) and δ­(CH3) at 1380 cm^–1^, absorption of amide III ν­(C–N) and δ­(N–H) at 1230 cm^–1^, and absorption of carbohydrate moieties ν­(C–O) and ν­(C–O–C) at 1080 cm^–1^. For the chondroitin sulfate spectra, the OH stretching vibration was dominated in the region above 2000 cm^–1^, the sulfate was seen around 1350 cm^–1^, and the S=O peaks at 1242 cm^–1^. The FT-IR spectra reveal that the chemical composition of the scaffolds remains highly conserved even after plasma treatment (Figure 3).

**Figure 3:**
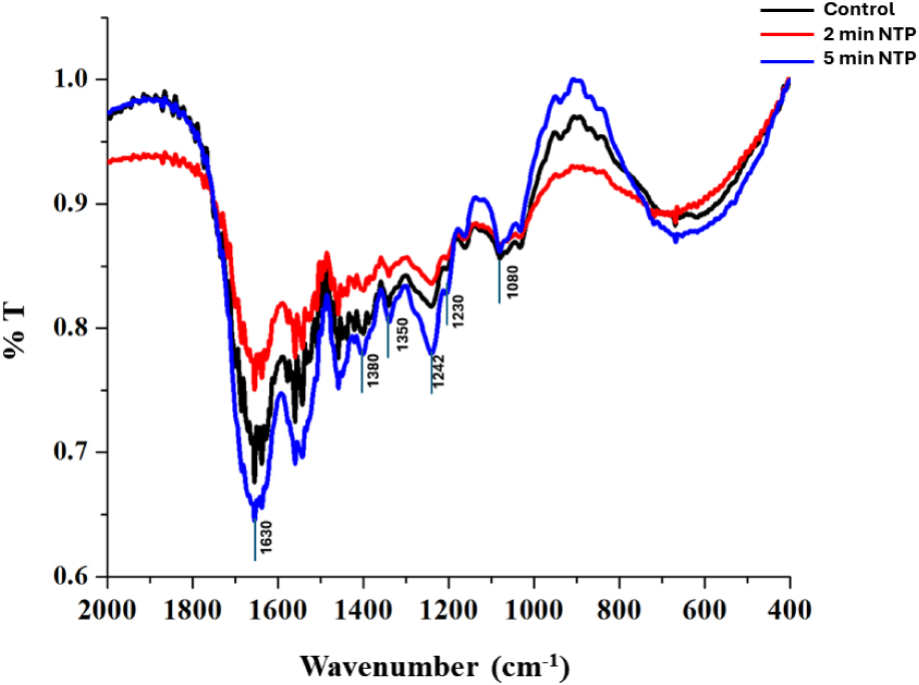
FTIR spectra of control, 2 and 5 min post-NTP treatment with a focus on the 2000–400 cm^–1^ region reveal that the chemical composition of the scaffolds remains highly conserved even after the NTP treatment (*y*-axis represents“%T”, which indicates percentage of transmittance).

The elemental composition analysis by EDS showed no significant changes in the levels of carbon, nitrogen, oxygen, and sulfur following NTP treatment of the scaffolds (results not shown).

### 3.4 Enhanced Hydrophilicity and Absorption Rates Following NTP Treatment

Untreated control scaffolds exhibit a contact angle of 106.7°, indicating a more hydrophobic surface as compared to NTP treatments. 2 min NTP showed greater hydrophilicity (contact angle 85.9°), and at 5 min, the NTP scaffold showed a reduction in contact angle as compared to the control (91.2°) (Figure 4).

**Figure 4:**
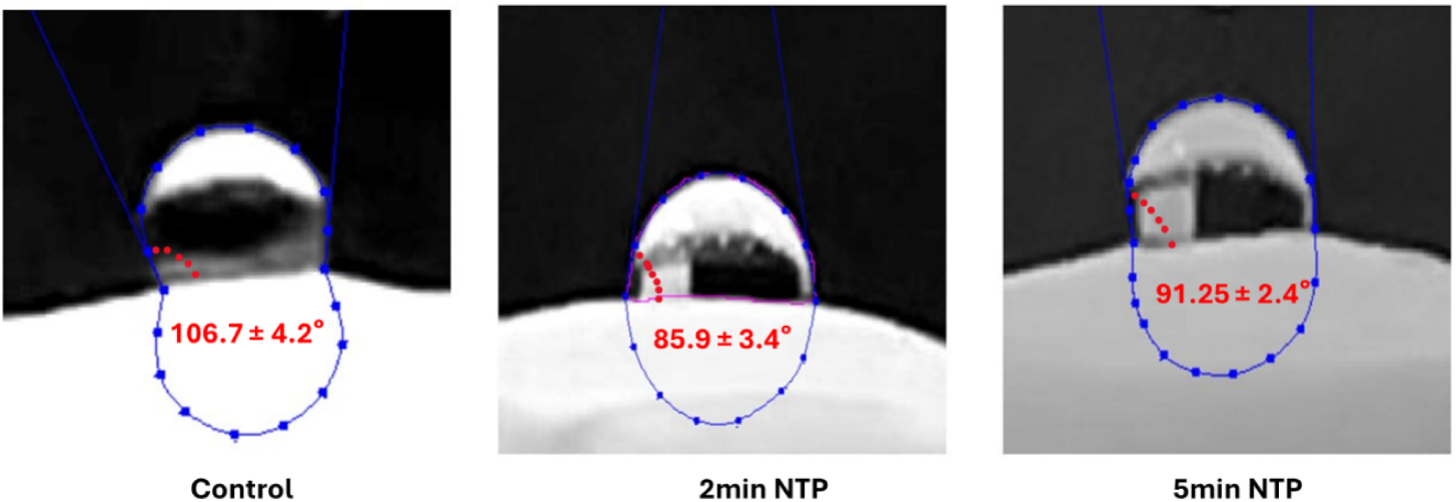
2 min NTP scaffold (85.9°) and 5 min (91.25°) NTP scaffold displayed a reduction in contact angle versus control (106.7°), indicating better wettability and greater hydrophilicity.

The liquid was absorbed completely in the untreated scaffold in ∼13 s (0.2 μL s^–1^), while both 2 and 5 min NTP scaffolds broadly showed a similar absorption rate of 30 μL s^–1^, with absorption of the given amount of liquid in 1/10th of a second.

### 3.5 Rate of Enzymatic Degradation of NTP-Treated Scaffolds

The degradation rate was significantly higher in 5 min NTP-treated scaffolds versus the control at all time points (*p* < 0.01). The 2 min treated scaffolds showed a similar degradation pattern compared to the control, as seen in Figure 5.

**Figure 5:**
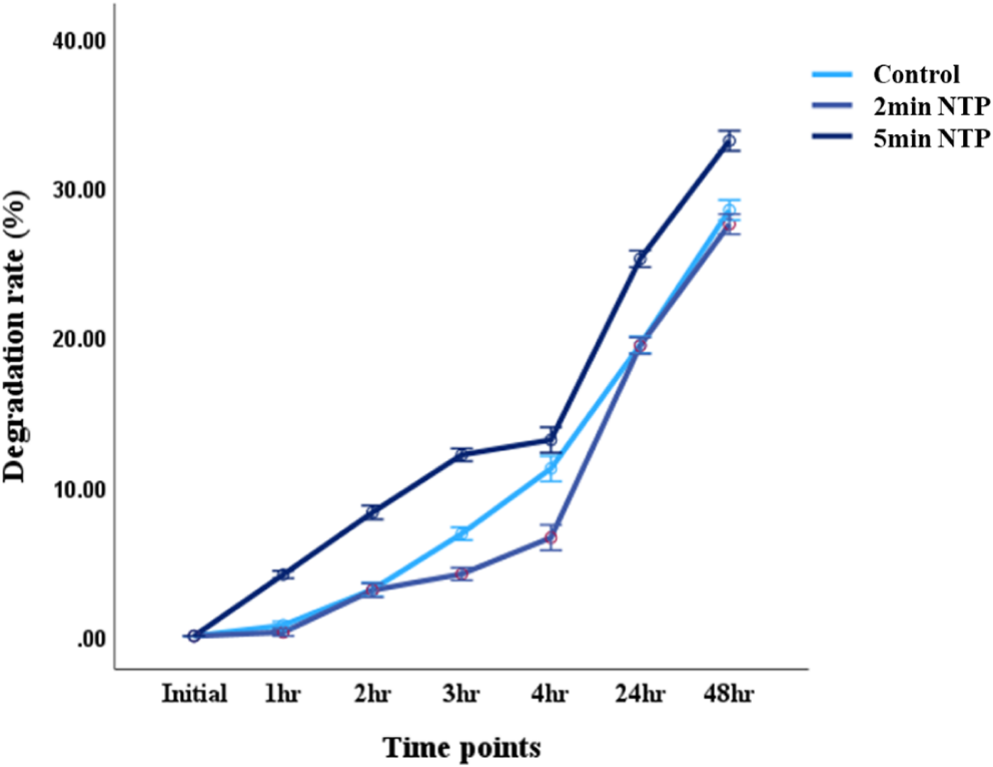
Enzyme Degradation profile of control, 2 and 5 min NTP CG scaffolds with the latter displaying a significantly higher degradation rate as compared to control at all time points.

### 3.6 Enhanced Cell Attachment and Increased Metabolic Activity on the NTP-Treated Scaffolds

Cell number increased following NTP treatment with a statistically significant increase observed after 2 min of NTP (*p* = 0.01) and 5 min of NTP (*p* = 0.02) when compared to the untreated control at day 5 as seen in DAPI staining (Figure 6 A). Similar trends were noted for viability, where all cell-seeded scaffolds remained metabolically viable following NTP treatment with a significant increase in cell viability noted in 2 min NTP at day 3 (*p* = 0.01). There was an increase in cell viability in 5 min NTP as well, compared to the control; however, this finding was not significant (Figure 6B).

**Figure 6:**
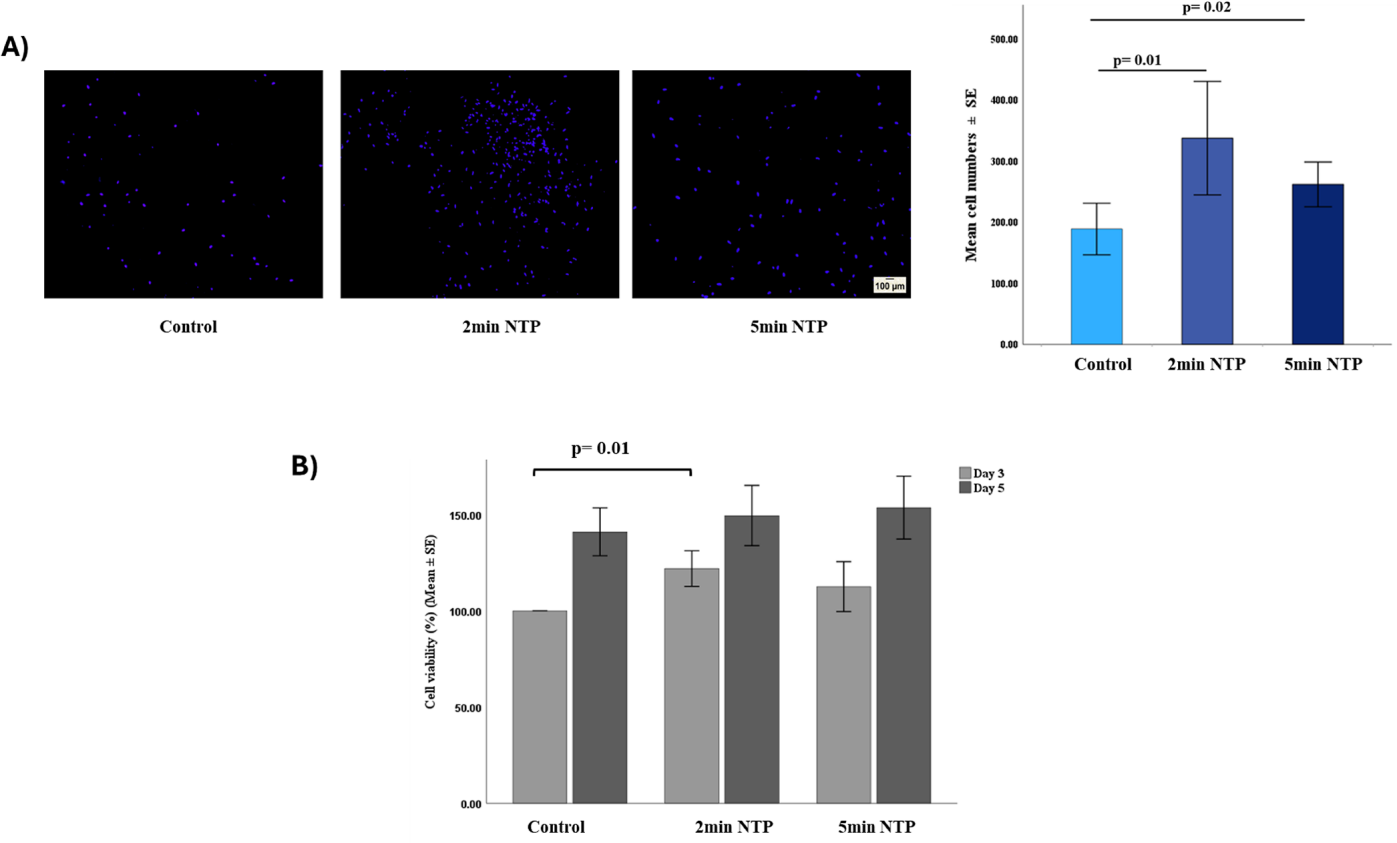
A) DAPI staining (*n* = 3) showed a significant increase in cell numbers following NTP treatment at 2 min (*p* = 0.01) and 5 min (*p* = 0.02); B) Cell viability after day 3 and day 5 of cell seeding on control, 2 and 5 min NTP-treated scaffolds. Cell viability was significantly higher on day 3 in 2 min NTP compared to control (*p* = 0.01).

### 3.7 NTP-Treated Scaffolds Support toward a Pro-Osteogenic Lineage

The increased calcium deposition during the late stage of osteogenic differentiation in NTP-treated collagen scaffolds compared to untreated controls was confirmed by alizarin red staining (Figure 7).

**Figure 7:**
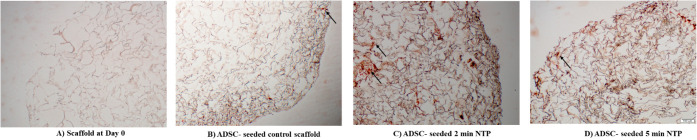
Day 21 alizarin red staining for calcium deposition: A) Scaffold at Day 0; B) ADSC- seeded control scaffold; C) ADSC-seeded 2 min NTP; D) ADSC-seeded 5 min NTP. Scale bar (100 μm).

The mRNA expression of osteogenic markers (RUNX2 and OCN) was assessed by quantitative RT-PCR on control and NTP scaffolds. RUNX2 showed a 9-fold increase at 2 and 5 min compared to the controls (*p* < 0.001). Similarly, the pro-maturation marker OCN expression was 2-fold higher in NTP-treated scaffolds (*p* < 0.001) compared to control or nonplasma-treated scaffolds (Figure 8).

**Figure 8:**
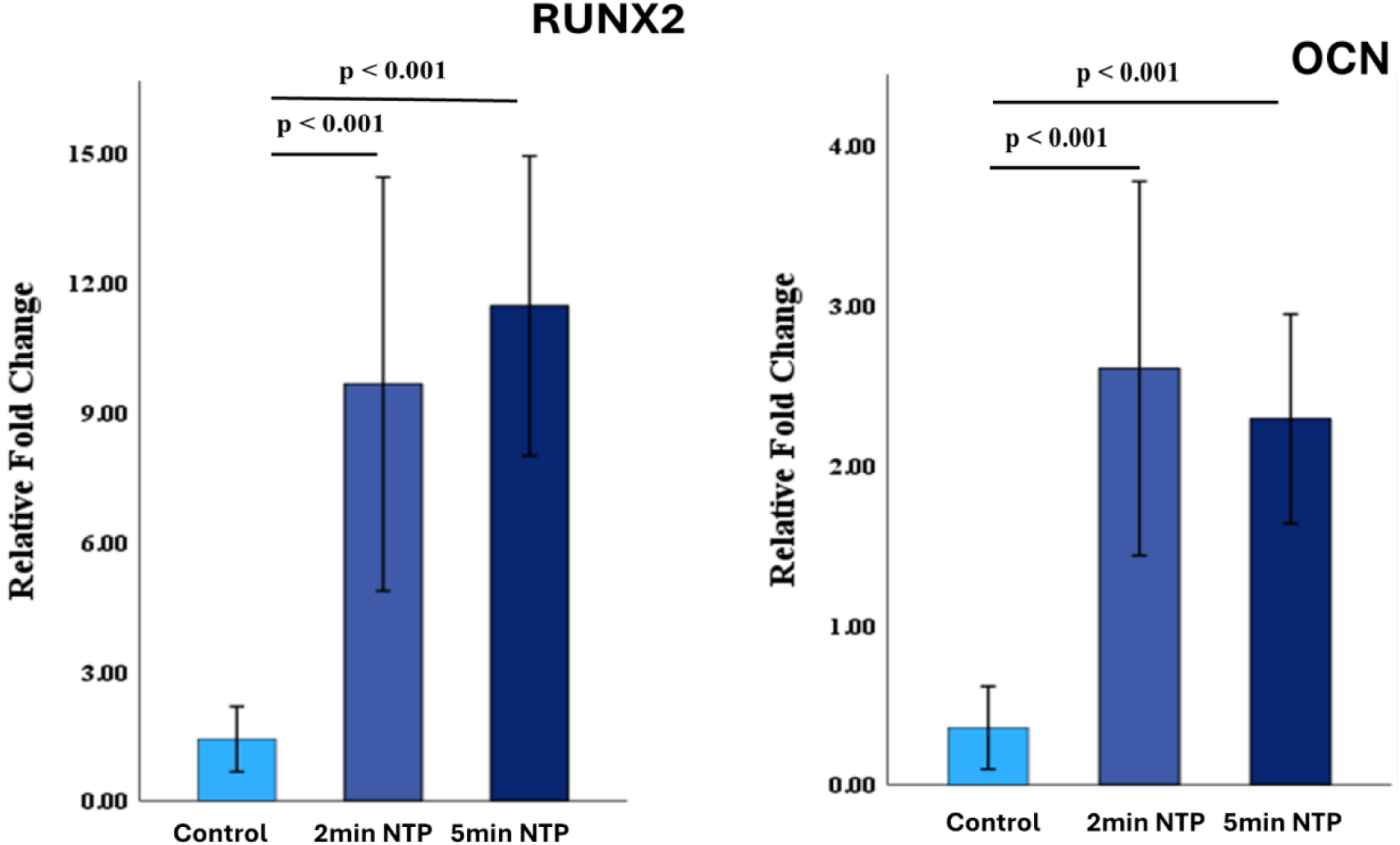
Relative fold change determined by quantitative real-time PCR (qRT-PCR) analysis of RUNX2 and OCN genes in the untreated scaffold and 2 and 5 min NTP-treated scaffold. There was a 9-fold increase in RUNX2 in the groups post-treatment (*p* < 0.001). Similarly, the late-stage osteogenic marker Osteocalcin showed a 2-fold higher in NTP-treated scaffolds (*p* < 0.01).

## 4 Discussion

Biocompatible and biophysical properties of scaffolds play an important role in the ability of cells to interact with, attach to, and grow on a biomaterial. While collagen scaffolds have excellent biocompatibility and are used clinically to treat various wounds, they often require additional cross-linking treatments to improve their biophysical features. Chemical cross-linking agents may require a 2-step process or can leave hazardous functional byproducts, which are not ideal for clinical use. In this study, we assessed an alternative cross-linking technique using nonthermal plasma (NTP) treatment on collagen scaffolds. We show that following NTP treatment, collagen scaffolds increase in mechanical stiffness and pore size, become more hydrophilic, have greater cell numbers, and support stem cell differentiation better than untreated controls.

Previous studies have also reported that NTP treatment modifies the biophysical characteristics of materials, which can influence cellular interaction by optimizing cell attachment surface area and maximizing the diffusion of nutrients in the scaffold; key parameters in tissue engineering.^1,38^ The SEM and histological analyses indicated extensive pore structure and wall thickness alteration following plasma treatment, which increases the mechanical stiffness of the scaffold. The increase in pore wall and strut thickness and pore area, especially in the 5 min treated sample, has been seen in other studies.^39^ It has been suggested that the rearrangement of the surface topography is due to the interaction and bombardment of high-energy ionized plasma particles onto the surface, which creates etches and grooves.^30^ This increase observed in the 5 min group can provide for better infiltration of cells and vasculature.^39^ Mozaffari et al. (2024) reported that nitrogen plasma treatment potentially leads to subtle reorientation of biomacromolecules on the gelatin nanofiber surface,^40^ which can be extended to explain the changes in the collagen fibers in our study (Figure 9). These minor orientation changes likely cause the collagen fibers to compact closer together while simultaneously increasing the pore size between them, resulting in thicker pore struts and enlarged pores with prolonged NTP exposure.

**Figure 9:**
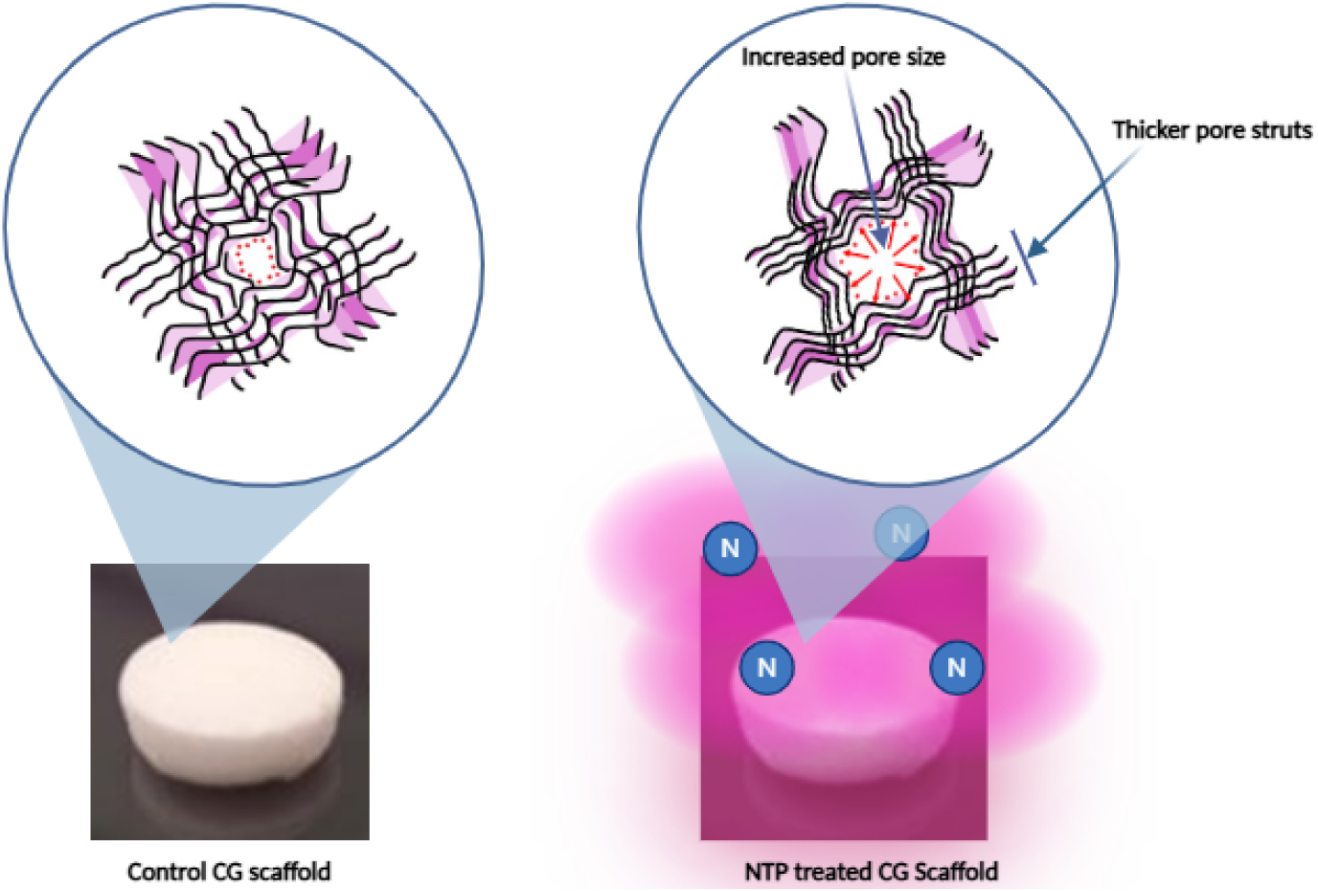
Proposed mechanism demonstrating how the NTP makes minor reorientation of biomacromolecules (collagen fibers), which leads to increased pore size and thicker wall struts in the CG scaffold following NTP treatment, which results in enhanced mechanical strength as well as improved cell migration, attachment, and growth.

Mechanical strength of the biomaterials tends to improve after the NTP treatment, with reports of up to 33% increase in strength.^41^ In this study, nitrogen NTP on a collagen scaffold showed about a 20% increase in the compressive modulus. This was due to surface cross-linking or densification effects induced by plasma, which provided better resistance against compressive stress.^42^

Importantly, the FT-IR analysis revealed that the major chemical functional groups of the CG scaffolds remained intact following plasma treatment. Our FT-IR results were similar to a previous study on oxygen NTP treatment of collagen films functionalized with chondroitin sulfate, which also found no detectable changes in functional groups.^16^ This indicates that while plasma does alter the scaffold pore morphology, it does not degrade or chemically compromise the bulk biomaterial. The retention of functional groups such as amide I and III, sulfate, and hydroxyl groups suggests that the scaffold still retains its native biochemical cues necessary for cellular interaction and ECM mimicry. Similarly, the elemental composition analysis of the control and treated scaffolds exhibited no significant change, indicating that the NTP preserves the inherent elemental characteristics of the scaffolds.

NTP has been employed widely for enhancing the wettability of biomaterials. Contact angle analysis revealed a reduction in water contact angles in the two NTP groups, with the highest reduction occurring in the 2 min of treatment (85.9°). Increased hydrophilicity and wettability are well-established driving factors for protein adsorption and cell adhesion, indicating greater biocompatibility, and the increase in hydrophilicity in combination with increased porosity and mechanical stiffness may be beneficial in increasing cell seeding density and cell attachment, which conforms with previous plasma treatment studies.^9,12,13,39^

Findings on degradation are consistent with those from the reports by Nyssanbek et al., 2024.^41^ The 2 min NTP scaffold retained resistance to degradation comparable to that of the untreated control, while the 5 min NTP scaffold did not. This implies that plasma treatment for brief durations would have a minimal effect on scaffold durability, but increased exposure would increase enzymatic accessibility due to microstructural modification. These findings underscore the significance of a balance between scaffold degradability and mechanical stability in the design of ECM-mimetic scaffolds. As shown above, improving the scaffold properties like wettability, porosity, and mechanical stiffness can increase cell attachment and growth. Plasma treatment has been reported to be an effective method to improve cell adhesion and tissue regeneration.^21,24,25,28,38^ Initial *in vitro* biocompatibility analysis in this study showed that 2 min NTP collagen-GAG scaffolds displayed enhanced ADSC attachment and viability on days 3 and 5. Optimal numbers of ADSCs and metabolic activity were found in the 2 min NTP group, which most likely resulted from synergistic effects of the optimal pore structure, hydrophilic nature, and intact biochemical integrity, promoting better biocompatibility. The slightly reduced viability in the 5 min group might reflect overmodification of the surface, which would disrupt maximum cell-scaffold interaction. These findings agree with earlier studies that moderate plasma treatment improves cellular performance, but prolonged time has diminishing advantages or could even be detrimental.^17,18,39^

The properties of plasma treatment in modifying surfaces and enhancing biomaterial stability have also enhanced the scaffold’s properties for bone regeneration.^43^ We tested the potential of the NTP scaffolds to support the cells toward the osteogenic lineage using alizarin red staining, detecting the presence of Ca^2+^ deposits in the groups that were supplemented with osteogenic media, indicating that the ADSCs differentiated to osteoblasts. NTP treatment promotes a greater osteogenic profile than untreated collagen scaffolds. RUNX2, the master transcription factor for osteoblast differentiation^44^ and OCN, a protein secreted by mature osteoblasts are key markers of osteogenic differentiation. Where RUNX2 is an early marker, OCN is a late marker of osteo-differentiation.^45,46^ RT-PCR results indicate that both RUNX2 and OCN were significantly upregulated in 2 and 5 min NTP scaffolds, indicating a higher rate of osteo-differentiation as compared to control or nonplasma-treated scaffolds.

The cell viability and differentiation in the NTP-treated scaffolds indicate that these constructs not only support cell survival but also actively promote osteogenic commitment, positioning them as promising candidates for bone regenerative applications. These findings align with previous studies on nonthermal plasma–modified chitosan and polymeric scaffolds, where such surface treatments are known to increase alkaline phosphatase activity, extracellular matrix mineralization, and osteogenic gene expression compared with untreated controls.^47^

While this study provides comprehensive insight into the short-term effects of nitrogen plasma treatment as a possible replacement for chemical cross-linking, further studies are warranted to evaluate long-term degradation, *in vivo* biocompatibility, and functional tissue integration. Future work should explore the molecular mechanisms by which NTP influences collagen-GAG interactions at the protein level and whether different gas compositions (e.g., argon, oxygen) may yield superior outcomes. Additionally, altering plasma setting as power and wavelength needs to be further explored.

## 5 Conclusion

In summary, this study demonstrates that NTP treatment effectively enhances the biophysical and biocompatible properties of collagen scaffolds while maintaining their chemical integrity. By enhancing scaffold stiffness, porosity, and hydrophilicity, NTP treatment promotes superior cell proliferation and osteogenic differentiation, key factors for successful tissue regeneration.

From a clinical translational perspective, these advances suggest that NTP-modified collagen scaffolds could offer a safer, more effective alternative to traditional cross-linking methods, with the potential to improve outcomes in regenerative therapies for bone and other connective tissues. The noninvasive nature and versatility of NTP technology further support its translational potential in the development of advanced biomaterials for personalized tissue engineering and regenerative medicine applications.

## Data Availability

All data generated or analyzed during this study are included in this published article.
